# Addressing filamentous fungi-related onychomycosis in the era of antifungal resistance: assessment of *Zataria multiflora* nanostructured lipid carrier topical gel in a double-blinded clinical trial

**DOI:** 10.22034/cmm.2024.345248.1582

**Published:** 2025-02-03

**Authors:** Maryam Moazeni, Hamidreza Kelidari, Armaghan Kazeminejad, Nasim Gholizadeh, Iman Haghani, Abolfazl Saravani, Shima Parsay, Yaser Nasirzadehfard, Ramin Mofarrah, Alireza Amini

**Affiliations:** 1 Invasive Fungi Research Center, Communicable Diseases Institute, Mazandaran University of Medical Sciences, Sari, Iran; 2 Department of Medical Mycology, School of Medicine, Mazandaran University of Medical Sciences, Sari, Iran; 3 Laboratory of Advanced Analysis in Biochemistry and Molecular Biology, Department of Biochemistry, Federal University of Rio de Janeiro, University City, Rio de Janeiro 21941-909, RJ, Brazil; 4 Department of Dermatology, Faculty of Medicine, Mazandaran University of Medical Sciences, Sari, Iran; 5 Student Research Committee, Mazandaran University of Medical Sciences, Sari, Iran; 6 Department of Medical Mycology, Imam Reza Hospital, School of Medicine, Ardabil University of Medical Sciences, Ardabil, Iran; 7 Department of Dermatology, Faculty of Medicine, Sari Branch, Islamic Azad University, Sari, Iran; 8 Social Security Organization, Vali-Asr Hospital, Ghaemshahr, Iran

**Keywords:** Clinical trials, Onychomycosis, Filamentous fungi, Nanostructured lipid carrier, *Zataria multiflora*

## Abstract

**Background and Purpose:**

Onychomycosis, a prevalent fungal infection affecting the nails, presents considerable difficulties in clinical treatment. *Zataria multiflora* (Zat), with its known antifungal properties, presents a promising approach for treatment. The present study focused on the evaluation of the effectiveness of Zat-NLC 1% gel against mold-associated onychomycosis, compared to a placebo.

**Materials and Methods:**

The Zat-loaded nanostructured lipid carriers (Zat-NLCs) were prepared and optimized by utilizing an ultrasonic probe approach. Antifungal susceptibility testing followed Clinical and Laboratory Standards Institute M38-A3 guidelines on the most common dermatophytes and non-dermatophytes fungal species. A double-blind trial with 40 participants (20 volunteers from each gender, equally divided into two groups, namely Zat-NLCs 1% gel and placebo receivers) evaluated Zat-NLC 1% gel efficacy. Causative agents were identified at the species level using a polymerase chain reaction-restriction fragment length polymorphism (PCR-RFLP) method.

**Results:**

A mono-dispersed suspension of spherical nanoparticles with zeta potential, Z-average, and polydispersity index of -26.6±7.7 mV, 273.9±3 nm, and 0.369±0.03, respectively, was achieved with no cytotoxicity. The Zat-NLCs demonstrated a significant inhibitory effect on both dermatophytes and non-dermatophyte fungal growth, *in vitro*. Effective improvement was observed in mycological criteria, compared to the placebo group (*P*<0.005) after 2 weeks of treatment. The mycological cure rate was 70% for Zat-NLCs gel after only 2 weeks. The results were notably different from those observed in the placebo group following the same duration of application (70% vs. 55%). However, the difference was insignificant in the mentioned groups after 4 weeks of application due to the prescription of routine antifungals for onychomycosis. The PCR-RFLP outputs revealed *T. mentagrophytes*/*interdigitale* complex and *A.* section Flavi as the predominant isolated species of dermatophytes and non-dermatophytes, respectively.

**Conclusion:**

Nanoscale colloidal systems loading with antifungals might be strongly considered a better and more efficient cure for mold-related dermatophytosis.

## Introduction

Onychomycosis caused by filamentous fungi is a common condition marked by the invasion of fungi into the nails, resulting in discoloration, thickness, and brittleness [ 1 ]. According to the statistics, its prevalence ranges from 2% to 14% in the general population, with greater incidence rates observed in the elderly (20% in those over 60 and 50% in those over 70) and males [ 2 ]. It presents a substantial therapeutic problem due to its chronic nature, risk of recurrence, and limited treatment options [ 3 ].

Building upon the challenges of treatment of onychomycosis, another significant obstacle is the slow rate of nail growth. Gradual replacement of the infected nail with a healthy one can prolong treatment time and frustrate patients as infected nails are slowly replaced by healthy ones [ 4 ]. Given the substantial concern about antimicrobial resistance and the global reports of terbinafine resistance being recognized as a significant factor, the need for novel antifungal therapeutic products becomes ever more apparent [ 3 ]. Usage of herbal medicinal extracts in designing new antifungal agents has attracted the consideration of many researchers due to its advantages, such as few side effects, ease of access, lack of resistance, and reasonable price [ 5 ].

Sustained drug delivery systems are increasingly being applied in regenerative medicine, particularly in antifungal agents, leading to a significant increase in their use [ 6 - 9 ]. Nanostructured lipid carriers (NLCs) have emerged as a viable method for topical antifungal therapy, overcoming limitations found in traditional formulations [ 5 , 10 ]. These lipid-based nanocarriers improve drug delivery by overcoming barriers, such as limited skin permeability, high dosage frequency, and low therapeutic efficacy.

The present study attempted to objectively assess the clinical symptoms and mycological findings associated with the topical administration of NLCs loaded with *Zataria multiflora* essential oils (Zat-EOs) gel 1% in the treatment of filamentous fungi-induced onychomycosis.

## Materials and Methods

### Ethical considerations

The present research was carried out in compliance with the ethical standards established in The Code of Ethics of the World Medical Association (Declaration of Helsinki) concerning studies involving human participants. The research protocol received endorsement from the Ethics Committee of Mazandaran University of Medical Sciences, identified by the reference number IR.MAZUMS.REC. 1401.14991.

Additionally, this study was registered with the Iranian Registry of Clinical Trials under the registration code IRCT20210611051539N3, which is available at https://www.irct.ir/. This registration promotes transparency and ensures that trial information is accessible. Informed consent was secured from all participants, who were made aware of their right to withdraw from the study at any point without incurring any adverse effects.

### Preparation and characterization of Zataria multiflora-loaded nanostructured lipid carriers

Materials used for Zat-NLCs were prepared according to the previously published article conducted by the team of the same researchers [ 5 ]. To fabricate Zat-NLCs, a modified ultrasonic probe method was applied based on previous research [ 5 ]. Physio-chemical characteristics of synthesized nanoparticles were assessed by Transmission Electron Microscope (TEM, Phillips CM 30 TEMm, Netherlands), the Malvern Zetasizer ZS (Nano ZA, Malvern Instruments, UK), and the photon correlation spectroscopy. The zeta potential and size distribution profile (polydispersity index PDI]) of the nanoparticles were also defined [ 5 ].

### Cell cytotoxicity and formulation of Zataria multiflora-loaded nanostructured lipid carriers 1% topical gel

Cell cytotoxicity of the fabricated product was evaluated earlier in the previous articles published by the current research team [ 11 ]. No adverse effects, such as skin irritation, rashes, or other allergic symptoms were observed after 30 days of Zat-NLCs gel 1% application [ 5 ]. The topical gel was also formulated according to the aforementioned study [ 5 ].

### In vitro antifungal susceptibility testing

Isolates of Aspergillus Sec. Flavi and Trichophyton mentagrophytes/interdigitale were used for the initial evaluation of the antifungal effect of Zat-NLCs. The two species were selected since they were the filamentous fungal species collected from patients with onychomycosis extensively. Each species comprised 10 species that had been isolated from the environment and also from patients inflicted with different types of dermatophytosis and Aspergillus-related diseases. The isolates were preserved in the reference culture collection of the Invasive Fungi Research Center located in Sari, Iran. They had previously undergone species-level identification through the sequencing of specific genes or regions, which included the internal transcribed spacer (ITS1-5.8s-ITS2) for T. mentagrophytes/interdigitale and B-tubulin for various Aspergillus species.

Antifungal susceptibility testing (AFST) was performed according to the modified M38-A3 document for filamentous fungi [ 12 ]. Along with Zat-NLCs, placebo (the vehicle without Zat-EOs), Zat-EOs, and also reverence antifungals (terbinafine for T. mentagrophytes/interdigitale and itraconazole for A. fumigatus) were used. The Zat-NLCs, Zat-EOs, and terbinafine/itraconazole were tested with concentrations within the ranges of 0.5% - 0.0001% (5000 - 1 µg/mL), 0.5% - 0.0001% (5000 - 1 µg/mL) and 16- 0.016 µg/mL, respectively. Paecilomyces variotii (ATCC 22319) was used as a reference strain to control the quality. Results were interpreted utilizing an inverted microscope (Motic AE31, Hong Kong, China).

### Clinical investigation of topical gels containing Zataria multiflora-loaded nanostructured lipid carriers

This study received ethical approval from the Ethics Committee of Mazandaran University of Medical Sciences under identification number IR.MAZUMS.REC. 1401.14991. Moreover, this research was registered with the Iranian Registry of Clinical Trials (IRCT) using the designated code IRCT20210611051539N3, which can be retrieved at https://www.irct.ir/.

A parallel clinical trial was conducted using a randomized, double-blind, placebo-controlled approach. Individuals with filamentous-related onychomycosis, who had not previously received antifungal therapy, participated in the research. This research included patients suffering from mild to moderate onychomycosis [ 13 ] who met both clinical and mycological criteria but excluded pediatric volunteers below 10 years old. The mycological criteria comprised successful cultures on Sabouraud Dextrose Agar (SDA) medium, along with a direct examination test utilizing 20% potassium hydroxide (KOH).

Groups A and B, each comprising 20 participants, consisted of the subjects treated with Zat-NLCs topical gel and placebo during this study, respectively. The patients were trained to apply Zat-NLCs gel twice a day between morning and evening.

For 1 month (30 days), the patients used topical Zat-NLCs gel formulation in conjunction with placebo. Their clinical and mycological parameters were assessed at three distinct time intervals, namely upon admission (baseline), after two weeks (day 15), and after four weeks (day 30). For the mycological criteria, negative results from KOH direct examination tests and cultures on the SDA medium were evaluated. Moreover, the overall approval of dermatologists was evaluated throughout the study. To assess the efficacy and safety of Zat-NLCs gel formulation in comparison with placebo after 2 and 4 weeks, data obtained from these assessments have been used.

### Molecular identification of the agents

Molecular assays were performed to confirm the results obtained from morphologic identification. Through a method previously described, genetic DNA was extracted from fresh colonies grown in the SDA medium [ 14 ]. The dermatophyte strains were identified at the species level by digestion of internal transcribed spacer (ITS1-5.8s-ITS2) region with *Mva*1 restriction enzyme. The species classified as *Aspergillus* were subsequently examined through the digestion of the tubulin gene utilizing *Alw*I (*BspP*I) (LifeTechnologies, Carlsbad, CA, USA) restriction enzyme. Polymerase chain reactions (PCR) were operated using universal primers ITS1 (5- TCCGTAGGTGAACCTGCGG-3) and ITS4 (5-TCCTCCGCTTATTG ATATGC–3), targeting the ITS rDNA region and Beta tubulin primers, namely Bt2a (5'-GGTAACCAAATCGGTGCTGCTTTC-3') and Bt2b (5-ACCCTCAGTGTAGTGACCCTTGGC-3), targeting the Beta tubulin gene.

To conduct the restriction fragment length polymorphism (RFLP) assay, the PCR products underwent digestion with restriction enzymes for a duration of 2 h at a temperature of 37 ºC [ 15 ,  16 ]. The PCR products and the configuration of the resulting digested fragments were analyzed using 1.5% and 2% agarose gels, respectively. Identification of the isolates was achieved by comparing the electrophoretic RFLP patterns against previously established profiles [ 15 , 16 ].

### Statistical analysis

The data assessment was conducted in SPSS software for Windows (version 22) created by SPSS Inc. (Chicago, IL, USA). The paired t-test was utilized to examine continuous variables within the groups, while the independent t-test was employed to assess continuous variables between the groups. For group comparisons in terms of categorical variables, the Chi-squared test or Fisher’s exact test was selected. Descriptive statistics were employed to evaluate the frequency as well as the demographic information. In addition, the McNemar test was implemented to analyze nonparametric paired data. A P value of less than 0.05 was considered statistically significant in all analyses.

## Results

### Characterization of synthesized Zataria multiflora-loaded nanostructured lipid carriers

**Figure 1:**
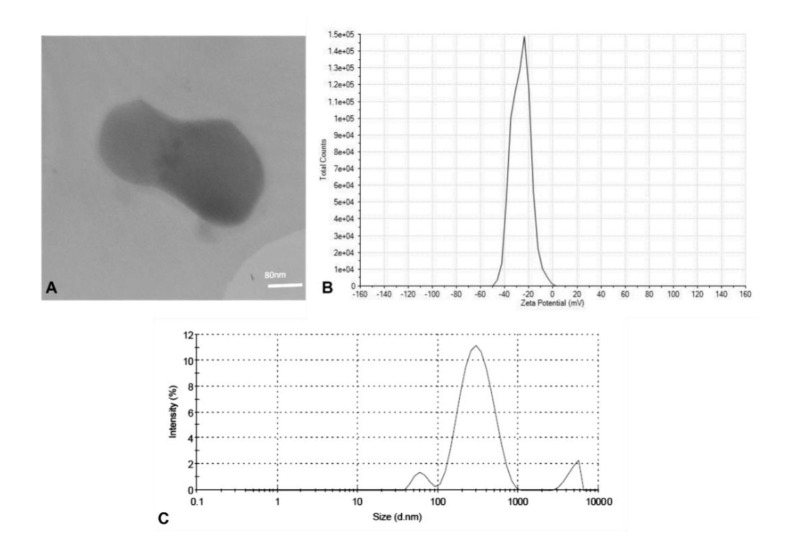
A) Sphere-shaped nanoparticles with an approximated size of 300 nm. Scale bar represents 60 nm. B) Zeta potential of Zat-NLCs suggesting the favorable steadiness of the nanoparticles. C) Particle size dispersal and PDI index graph.

Mono-dispersed nanoparticles were fabricated comprising 0.7% stearic acid, 0.3%, Oleic acid, and 1.8% Span 80+ 2.8% Tween 80 as solid lipid phase (w/w), liquid lipid phase (w/w), and emulsifiers (w/w), respectively. The suspension exhibited a Z-average of 273.9±3 nm, a PDI index of 0.369±0.03, and a zeta potential of -26.6±7.7 mV. A large portion of the suspension (88.6%) comprised nanoparticles with an estimated size of 300 nm. The TEM analysis indicated the presence of spherical particles with soft surfaces. Properties of the synthesized Zat-NLCs are presented in Figure 1.

The particle size exactly impacts the occurrence of flocculation or precipitation. Usually, the particle dimensions of NLCs span 150-300 nm [ 17 ,  18 ]. For electrostatic stability, the nanoparticles should have a zeta potential of less than -30 mV or greater than +30 mV [ 19 ]. The zeta potential data along with particle size distribution showed that the method of fabrication suggested in this research met the requirements for efficient production of the nanoparticles.

### In vitro antifungal susceptibility testing

Results of AFST were analyzed in accordance with Clinical and Laboratory Standards Institute standards applicable to *Aspergillus* and dermatophyte species. The minimum inhibitory concentration (MIC) ranges were 0.031-0.004 8 μg/mL and 0.25-0.125 μg/mL for *T. mentagrophytes*/*interdigitale* and *A.* sec. Flavi species against terbinafine and itraconazole, respectively. In the case of Zat-EOs, the MIC ranges were achieved as 40-160 μg/mL and 620-1250 μg/mL for *T. mentagrophytes/interdigitale* and *A. flavus*, respectively. Compared to Zat-EOs results, the findings revealed a notable reduction in MIC ranges when the fungus was exposed to Zat-NLCs (*P*<0.005) (Table 1).

**Table 1:** Geometric mean, MIC_50_, and MIC_90_ values were determined by assessment of the susceptibility of the most commonly isolated species against Zat-EOs, Zat-NLCs, and also reference antifungals.

	Terbinafine (µg/mL)	*Zataria multiflora* essential oils µg/mL (%)	*Zataria multiflora*-nanostructured lipid carriers µg/mL (%)		Itraconazole (µg/mL)	*Zataria multiflora* essential oils µg/mL (%)	*Zataria multiflora*-nanostructured lipid carriers µg/mL (%)
*Trichophyton mentapgophytes*/*Interdigitale* (n=10)	0.008	80 (0.008)	10 (0.001)	*Aspergillus* section *Flavi* (n=10)	0.25	620 (0.062)	310 (0.031)
	0.008	80 (0.008)	10 (0.001)		0.25	620 (0.062)	160 (0.016)
	0.004	80 (0.008)	10 (0.001)		0.125	1250 (0.125)	310 (0.031)
	0.008	40 (0.004)	10 (0.001)		0.25	620 (0.062)	310 (0.031)
	0.004	40 (0.004)	10 (0.001)		0.25	1250 (0.125)	620 (0.062)
	0.016	40 (0.004)	10 (0.001)		0.25	1250 (0.125)	620 (0.062)
	0.031	80 (0.008)	10 (0.001)		0.125	1250 (0.125)	620 (0.062)
	0.031	80 (0.008)	10 (0.001)		0.125	620 (0.062)	310 (0.031)
	0.004	160 (0.016)	20 (0.002)		0.25	1250 (0.125)	310 (0.031)
	0.008	160 (0.016)	40 (0.004)		0.125	1250 (0.125)	620 (0.016)
MIC_50_*	0.008	80	10	MIC_50_	0.25	1250	310
MIC_90_	0.031	160	38	MIC_90_	0.25	1250	620
GM	0.0091	74.6426	12.3114	GM	0.1895	944.2841	334.3662
		*P* value				*P* value	
		0.0006				0.0001	

* **MIC:** Minimum inhibitory concentration, **MIC_50_:** minimal concentration that inhibits 50% of isolates; **MIC_90_:** minimal concentration that inhibits 90% of isolates, **GM:** Geometric mean

### Molecular identification of the etiological agents

In total, the findings derived from the RFLP assay indicated *Aspergillus* sec. Flavi is the most predominant species (n=21, 52.5%) followed by *Aspergillus* sec. Nigri (n=7, 17.5%), *Aspergillus* sec. Terrei (n=1, 2.5%), and *Aspergillus* sec. Fumigati (n=1, 2.5%). The *Alw*1 restriction enzyme was not able to identify 5 (12.5%) *Aspergillus* isolates which were then categorized as “not defined” *Aspergillus* species. Among dermatophytes, *T. mentagrophytes*/*interdigitale* was the only identified species that was responsible for 5 (12.5%) of the onychomycosis cases here.

These results align with those of earlier research conducted in Iran, such as the study performed by Motamedi et al., who also reported dermatophytes as the prevalent etiologic agents of onychomycosis (35.8%) followed by yeast and non-dermatophytes filamentous fungi. Based on their findings, *Aspergillus* was the most commonly reported species [ 20 ]. Similarly, Afshar et al. conducted a study on the molecular epidemiology of onychomycosis in the North of Iran and described parallel results, with *Aspergillus* species being the most prevalent agents among non-dermatophytes-related onychomycosis. However, *T. mentagrophytes*/ *interdigitale* was reported as the most predominant agent of onychomycosis associated with dermatophytes [ 21 ]. In the present study, the isolated fungal species were identified by PCR-RFLP assay to the section/complex level. However, to establish a relationship between the species and the effectiveness of the new formulation, the number of dermatophytes among all isolated filamentous fungi was insufficient.

### Assessment of clinical ending

In total, 40 participants were enrolled in this study, with an equal distribution of genders. The participants were divided into two groups: one group received Zat-NLCs 1% gel, while the other group received a placebo. Regarding age, no notable differences were detected among the various age groups; however, individuals aged 20-30 years old constituted the greatest portion of participants (27.5%). In terms of occupation, housewives were more impacted, compared to other occupations. Moreover, individuals in other occupations did not exhibit significant differences. Additionally, toenails were reported to be more affected by filamentous fungi, primarily saprophyte fungi (mainly *Aspergillus* species). Comprehensive demographic data of the participants are summarized in Table 2.

**Table 2:** Demographic data of 40 participants.

		Total (%)
Gender	Female	30 (75%)
	Male	10 (25%)
Age (years)	20-30	11 (27.5%)
	31-40	8 (20%)
	41-50	9 (22.5%)
	51-60	5 (12.5%)
	>60	7 (17.5%)
Occupation	Accountant	1 (2.5%)
	Computer Engineer	1 (2.5%)
	Physician	1 (2.5%)
	Farmer	2 (5%)
	Freelance	3 (7.5%)
	Hair Stylist	4 (10%)
	Housewife	21 (52.5%)
	Nurse	3 (7.5%)
	Police	1 (2.5%)
	Teacher	2 (5%)
	Welder	1 (2.5%)
Site of infection	Fingernail	12 (30%)
	Toenail	28 (70%)
Isolated fungal species	*Aspergillus* section Flavi	21 (52.5%)
	*Aspergillus* section Fumigati	1 (2.5%)
	*Aspergillus* section Nigri	7 (17.5%)
	*Aspergillus* section Terrei	1 (2.5%)
	*Aspergillus* species (Not defined)	5 (12.5%)
	*Trichophyton mentagrophytes*/*interdigitale*	5 (12.5%)
Underlying diseases	DM	5
	HT	3
	DLP	2
Dermatologist diagnosis	Non-dermatophyte Onychomycosis	36 (90%)
	Tinea unguium	4 (10%)

DM: Diabetes Mellitus, HT: Hypertension, DLP: Dyslipidemia

Administration of Zat-NLCs 1% gel therapy resulted in a meaningful improvement in terms of the approval of the dermatologist even after two weeks of application, compared to placebo. However, in the case of nail appearance, neither Zat-NLCs gel nor placebo application resulted in any improvement. Nevertheless, the fungal culture test, which is the gold standard diagnosis of proven onychomycosis, was negative after 2 weeks of Zat-NLCs 1% gel application (18 negatives out of 20 cases, 90%) (*P*=0.001). However, for the same period of time, the placebo application did not yield significant results (11 negative out of 20 cases, 55%) (*P*=0.031).

The hypothesis was supported by the evaluation of mycological criteria, which consisted of direct examination and culture results, in male and female patients who received Zat-NLC gel and placebo for 2 and 4 weeks. Following a two-week treatment period, a notably greater proportion of patients treated with Zat-NLC 1% gel exhibited negative outcomes in laboratory assessments, such as direct KOH examination and culture, compared to the placebo group. The mycologic cure rates were documented at 70% and 55% for the Zat-NLC and the placebo groups, respectively.

Similarly, a significant number of participants showed negative results on both direct examination and culture after 4 weeks of treatment and even placebo. These outcomes revealed the effective role of itraconazole in routine therapy. However, the effectiveness of Zat-NLC gel was observed only after two weeks of prescription. According to Table 3, the most notable enhancement was observed after 2 weeks of Zat-NLC topical gel application.

**Table 3:** Results of Zat-NLCs and placebo study groups after 2 and 4 weeks of intervention.

Results of mycologic criteria	Negative results of microscopic examination (%)				Negative results of culture (%)				Cured cases based on dermatologist approval (%)			
	Zat-NLCs		Placebo		Zat-NLCs		Placebo		Zat-NLCs		Placebo	
	Duration of medication (weeks)											
	2	4	2	4	2	4	2	4	2	4	2	4
	14 (70%)	18 (90%)	11 (55%)	20 (100%)	18 (90%)	20 (100%)	11 (55%)	20 (100%)	14 (70%)	14 (70%)	9 (45%)	9 (45%)
*P* value	0.000	0.000	0.000	0.000	0.001	0.000	0.031	0.000	0.001	0.000	0.125	0.031

## Discussion

Onychomycosis has a reinfection rate of 20-25% following successful treatment and a recurrence rate of 6.5-53% [ 13 ]. Therefore, healthcare providers should consider the ethical implications, financial burden, adherence rate, and side effects associated with each therapeutic modality before selection and justification of a treatment for patients. For many patients, onychomycosis can pose both a health concern and a cosmetic issue, potentially resulting in psychological burdens for patients and their families. Given the generally benign course of the disease, healthcare professionals should inform patients that onychomycosis is still an infection that can spread to other areas of the body and other family members. Lack of this awareness could contribute to the emergence of drug-resistant strains and, consequently, the failure of effective treatment.

Most effective cure for moderate to severe onychomycosis usually is oral therapy which is considered the gold standard for onychomycosis management [ 22 ]. The leading oral agents are terbinafine, itraconazole, and fluconazole. However, oral therapy demands extended usage due to its limited bioavailability and also inadequate ability to sustain enough drug concentrations in the nail bed [ 22 ]. Moreover, side effects of oral medications, including drug-drug interactions, hepatotoxicity, and congestive heart failure are important concerns due to their systemic absorption [ 22 ].

When fungi infiltrate a substrate, it is probable that an external biofilm, referred to as the extracellular matrix, will develop (ECM) [ 23 ]. The ECM enables biofilms to resist the immune response of the host and inhibit the antifungal agents penetrating the nail bed [ 24 ]. Hence, the number of Food and Drug Administration-certified topical antifungal agents has been limited [ 25 ]. All topical prescriptions are advised for mild to moderate diseases and the suggested duration of treatment is above 48 weeks [ 22 ]. Therefore, not only the efficacy of topical antifungals is a challenge, but also the reduction of the period of treatment is an important issue that needs to be updated.

Addressing onychomycosis in the time of antifungal resistance is the other unresolved issue that needs more consideration. Importance of antifungal stewardship and the application of topical antifungal treatments are two notable issues that highlight the possible application of herbal medicine in the management of fungi-associated nail diseases.

Among herbs, *Zataria multiflora* Boiss (Shirazi thyme), which belongs to the *Lamiaceae* family and is endemic mainly to Afghanistan, Iran, and Pakistan, has recently received great attention. Previous studies have investigated the antifungal effects of *Z. multiflora* on various fungal species [ 26 , 27 ]. Fard et al. documented the efficacy of Zat and terbinafine in improving and treating skin dermatophytosis [ 5 ].

Another study conducted by the same research group that performed the present study revealed the significant antifungal effect of Zat on Candida-related onychomycosis only after 2 weeks of application [ 10 ] with a mycological cure rate of 80%.

Extract of *Z. multiflora* comprises a variety of monoterpenoids, including thymol, carvacrol, and p-Cymene and is used conventionally as a food additive, antiseptic, and diuretic as well as for pharmacological and cosmetic goals [ 28 ]. Nevertheless, the evaporation of the ingredients and the effective entrance of the agent into the nail bed creates a challenge.

Nanoparticles, especially NLCs, provide a solution by improving drug penetration and ensuring sustained release [ 6 ,  9 ,  29 ]. The NLC nanoparticles can penetrate the outermost layer of the skin more effectively, which improves drug absorption and its entry into the skin. This leads to a better therapeutic response, as shown in earlier studies [ 30 ,  31 ]. The primary advantage of these lipid carriers lies in their ability to facilitate more rapid drug absorption into the epidermis. [ 32 ]. Previous research has demonstrated that the combination of lipid nanoparticles with skin lipids facilitates the exchange of drugs. These lipid nanoparticles serve as a sophisticated delivery system, capable of transporting medications to specific target sites [ 33 ].

In the present study, the duration of treatment had an impact on the advancement of the disease, achieving a mycological cure rate of 70% after two weeks of Zat-NLC gel application. A combination of itraconazole with this innovative treatment method results in a notably enhanced treatment response.

## Conclusion

The current study was conducted upon research into the application of NLC nanoparticles, aiming to explore their potential in improving treatment effectiveness and shortening treatment duration. By combination of itraconazole with Zat-NLCs in compliance with ethical standards, the recovery time was notably decreased. This study revealed promising results on the potent antifungal properties of *Z. multiflora* when encapsulated in NLCs, offering a swift and effective treatment alternative for onychomycosis caused by filamentous fungi. Nevertheless, further research is necessary to evaluate the usefulness of these antifungal agents independently, without the need for standard medication.

## References

[1] Lipner SR, Scher RK ( 2019). Onychomycosis: Clinical overview and diagnosis. J Am Acad Dermatol.

[2] Falotico JM, Lipner SR ( 2022). Updated perspectives on the diagnosis and management of onychomycosis. Clin, Cosmet Investig Dermatol.

[3] Gupta AK, Taborda VB, Taborda PR, Shemer A, Summerbell RC, Nakrieko K-A ( 2020). High prevalence of mixed infections in global onychomycosis. PLoS One.

[4] Vlahovic TC ( 2016). Onychomycosis: evaluation, treatment options, managing recurrence, and patient outcomes. Clin in podiatr med and surg.

[5] Fard YN, Kelidari H, Kazeminejad A, Mousavi SJ, Hedayati MT, Mosayebi E, et al ( 2023). Enhanced treatment in cutaneous dermatophytosis management by Zataria multiflora-loaded nanostructured lipid carrier topical gel: A randomized double-blind placebo-controlled clinical trial. J Drug Deliv Sci Technol.

[6] Kelidari HR, Moazeni M, Babaei R, Saeedi M, Akbari J, Parkoohi PI, et al ( 2017). Improved yeast delivery of fluconazole with a nanostructured lipid carrier system. Biomed Pharmacother.

[7] Moazeni M, Davari A, Shabanzadeh S, Akhtari J, Saeedi M, Mortyeza-Semnani K, et al ( 2021). In vitro antifungal activity of Thymus vulgaris essential oil nanoemulsion. J Herbal Med.

[8] Moazeni M, Kelidari HR, Babaei R, Gholami S, Nabili M, Gohar AA ( 2016). Solid lipid nanoparticles as an effective carrier of voriconazole to overcome the resistant isolates of Aspergillus fumigatus. Curr Med Mycol.

[9] Moazeni M, Kelidari HR, Saeedi M, Morteza-Semnani K, Nabili M, Gohar AA, et al ( 2016). Time to overcome fluconazole resistant Candida isolates: solid lipid nanoparticles as a novel antifungal drug delivery system. Colloids Surf B: Biointerfaces.

[10] Moazeni M, Kelidari H, Nasirzadehfard Y, Shokohi T, Roohi B, Hajheidari Z, et al ( 2024). Lesson from nature: Zataria multiflora nanostructured lipid carrier topical gel formulation against Candida-associated onychomycosis, a randomized double-blind placebo-controlled clinical trial. Med Drug Disco.

[11] Kelidari HR, Moemenbellah-Fard MD, Morteza-Semnani K, Amoozegar F, Shahriari-Namadi M, Saeedi M, Osanloo M ( 2021). Solid-lipid nanoparticles (SLN) s containing Zataria multiflora essential oil with no-cytotoxicity and potent repellent activity against Anopheles stephensi. J Parasitic Dis.

[12] Institute CLS ( 2008). Reference method for broth dilution antifungal susceptibility testing of filamentous fungi; approved standard, 2nd ed. CLSI document M38-A2.

[13] Yousefian F, Smythe C, Han H, Elewski BE, Nestor M ( 2024). Treatment Options for Onychomycosis: Efficacy, Side Effects, Adherence, Financial Considerations, and Ethics. J Clin Aesthet Dermatol.

[14] Abastabar M, Mirhendi H, Rezaei‐Matehkolaei A, Shidfar MR, Kordbacheh P, Makimura K ( 2014). Restriction analysis of β‐tubulin gene for differentiation of the common pathogenic dermatophytes. J Clin Lab Anal.

[15] Nasri T, Hedayati MT, Abastabar M, Pasqualotto AC, Armaki MT, Hoseinnejad A, Nabili M ( 2015). PCR-RFLP on β-tubulin gene for rapid identification of the most clinically important species of Aspergillus. J Microbiol Methods.

[16] Rezaei-Matehkolaei A, Makimura K, Shidfar M, Zaini F, Eshraghian M, Jalalizand N, et al ( 2012). Use of single-enzyme PCR-restriction digestion barcode targeting the internal transcribed spacers (ITS rDNA) to identify dermatophyte species. Iran J Public Health.

[17] Junyaprasert VB, Teeranachaideekul V, Souto EB, Boonme P, Müller RH ( 2009). Q10-loaded NLC versus nanoemulsions: Stability, rheology and In vitro skin permeation. Int J pharm.

[18] Yoon G, Park JW, Yoon I-S ( 2013). Solid lipid nanoparticles (SLNs) and nanostructured lipid carriers (NLCs): recent advances in drug delivery. J Pharm Investig.

[19] Thatipamula R, Palem C, Gannu R, Mudragada S, Yamsani M ( 2011). Formulation and In vitro characterization of domperidone loaded solid lipid nanoparticles and nanostructured lipid carriers. Daru.

[20] Motamedi M, Ghasemi Z, Shidfar M, Hosseinpour L, Khodadadi H, Zomorodian K ( 2016). Growing incidence of non-dermatophyte onychomycosis in Tehran, Iran. Jundishapur J Microbiol.

[21] Afshar P, Khodavaisy S, Kalhori S, Ghasemi M, Razavyoon T ( 2014). Onychomycosis in north-East of Iran. Iran J Microbiol.

[22] Gupta AK, Mays RR, Versteeg SG, Shear NH, Piguet V ( 2018). Update on current approaches to diagnosis and treatment of onychomycosis. Expert Rev Anti Infect Ther.

[23] Gupta AK, Daigle D, Carviel JL ( 2016). The role of biofilms in onychomycosis. J Am Acad Dermatol.

[24] Kernien JF, Snarr BD, Sheppard DC, Nett JE ( 2018). The interface between fungal biofilms and innate immunity. Front immunol.

[25] Elkeeb R, Hui X, Murthy N, Maibach HI ( 2014). Emerging Topical Onychomycosis Therapies–Quo Vadis?. Expert Opin Emerg Drugs.

[26] Gonoudi E, Rezai M, Farrokhnia T, Goudarzi M, Sima A ( 2021). Comparison of antifungal efficacy of Zataria multiflora and nystatin for treatment of denture stomatitis: A randomized clinical trial. J Dent.

[27] Monfared AA, Yazdanpanah M, Zareshahrabadi Z, Pakshir K, Ghahartars M, Mehrabani D, et al ( 2021). Chemical composition and antifungal activities of aromatic water of Zataria multiflora Boiss. Curr Med Mycol.

[28] Sajed H, Sahebkar A, Iranshahi M ( 2013). Zataria multiflora Boiss.(Shirazi thyme)—an ancient condiment with modern pharmaceutical uses. J Ethnopharmacol.

[29] Kelidari HR, Babaei R, Nabili M, Shokohi T, Saeedi M, Gholami S, et al ( 2018). Improved delivery of voriconazole to Aspergillus fumigatus through solid lipid nanoparticles as an effective carrier. Colloids Surf A Physicochem Eng Asp.

[30] Doktorovova S, Souto EB ( 2009). Nanostructured lipid carrier-based hydrogel formulations for drug delivery: a comprehensive review. Expert Opin Drug Deliv.

[31] Souto E, Müller R ( 2008). Cosmetic features and applications of lipid nanoparticles (SLN®, NLC®). Int J Cosmet Sci.

[32] Stewart PL ( 2017). Cryo‐electron microscopy and cryo‐electron tomography of nanoparticles. Wiley Interdiscip Rev Nanomed Nanobiotechnol.

[33] Mahant S, Rao R, Souto EB, Nanda S ( 2020). Analytical tools and evaluation strategies for nanostructured lipid carrier-based topical delivery systems. Expert Opin Drug Deliv.

